# Diagnosing Latent Tuberculosis Infection in the HIV Era

**DOI:** 10.2174/1874306400802010052

**Published:** 2008-05-23

**Authors:** Philippe H Lagrange, Jean Louis Herrmann

**Affiliations:** 1 Service de Microbiologie, Hôpital Saint Louis, Paris, France; 2 Service de Microbiologie, Hôpital Raymond Poincaré, Garches, France

**Keywords:** Tuberculin skin test, Interferon-gamma release assays, HIV-infected, latent tuberculosis infection, active tuberculosis, whole-blood killing assay, ELISA, antibody, PGL-Tb1.

## Abstract

Tuberculin skin testing (TST) and Interferon-gamma (IFNγ)release assays (IGRAs) are presently the only available assays for the detection of *Mycobacterium tuberculosis* infected individuals. IGRAs might progressively replace TST, as numerous published reports establish their higher specificity and similar sensitivity when tested in BCG vaccinated, immunocompetent individuals or in populations who may have been in contact with atypical mycobacteria. However, few published reports have commented on their role in TB diagnosis in immunocompromised individuals (HIV, immunosuppressive therapy, cancer…). It is the purpose of this report to review IGRAs published studies in HIV individuals in endemic and non endemic area for tuberculosis (TB). IGRAs were tested in the presence or absence of active TB but correlated to duration of exposure. In newly diagnosed active TB, IGRAs demonstrated a similar sensitivity to TST. In TB non infected individuals, TST and IGRAs also gave similar values when categorization of individuals was correlated to the risk of infection. A higher number of positive IGRAs was observed in individuals from TB endemic areas, in similar proportions to immunocompetent individuals. Comparison between the two IGRAs: QuantiFERON-TB Gold^®^ (QF-TB, Cellestis, Australia) and T-SPOT-TB^®^ (Oxford Immunotec, UK), and against TST, in the same HIV population demonstrates a higher sensitivity of T-SPOT-TB and TST than QF-TB. Indeterminate results, which correspond to the absence of a positive T-cell IFNγ response towards phytohemaglutinin (PHA), is a key point when comparing both IGRAs. This PHA control is indicative of the level of immunosuppression observed in the tested individual. QF-TB seems to present, in HIV populations, more indeterminate results than T-SPOT-TB. The calibration and/or concentration of PBMC on nitrocellulose membrane for the T-SPOT-TB, as compared to a whole blood assay, might explain this difference, with less indeterminate results with the T-SPOT-TB assay. Neither assay is able to differentiate active TB from latent TB infection (LTBI). Several laboratories have tried new antigenic epitopes to solve this issue. It is of importance that these studies need to be repeated on a larger scale by others to validate their results. Two blood assays might add information characterising the evolution from LTBI to active TB: either by losing protective immunity, as demonstrated by the whole blood killing assay, or by evaluating the kinetics of the antibodies synthesized against *M. tuberculosis* specific antigens. In conclusion, longitudinal studies are still needed to validate IGRAs and other assays and to define their respective predictive values.

## INTRODUCTION

In most individuals infection with *Mycobacterium tuberculosis* is contained by the host’s immune defences and the infection remains latent [1,2]. The World Health Organisation (WHO) has estimated that approximately a third of the world’s population is infected with *M. tuberculosis*, the vast majority with Latent Tuberculosis Infection (LTBI) [3]. The *M. tuberculosis* bacilli that persist in symptom-free immunocompetent individuals with LTBI, can reactivate however, and cause active TB disease in about 10% of those infected over a lifetime [4]. Currently, it is difficult to predict exactly when and who among the latently infected individuals will develop the disease. Targeted testing and treatment for LTBI in high risk populations is a key component of TB control in many low-incidence, high income countries as a consequence of this risk of progression from LTBI to active TB disease [5].

The rational of such preventive treatment is also linked to TB transmission in the community occurring mostly before a diagnosis of TB is made in the index case, even when an optimal TB control program is in place: new and undiagnosed cases are the driving force behind the current TB epidemic [6]. It has been calculated that if each new TB case infected up to 10 susceptible contacts before diagnosis, up to 40 million new infections might occur worldwide every year, adding to the pool of existing LTBI [7]. Implementation of an effective screening program in high risk populations that would identify individuals with LTBI and treat them to prevent disease would be of enormous operational value.

The determinants for increased risk of progression from LTBI to active TB disease are a recent infection with *M. tuberculosis* and several host-related factors, all of which seems to be associated with an impaired cell-mediated immune response [8]. Among this high risk populations, individuals infected with the Human Immunodeficiency Virus (HIV) are the most prone to reactivate the persistent *M. tuberculosis* bacilli. Likewise, after a primary infection, the risk of developing active TB disease in HIV-positive individuals is increased many fold, even when antiretroviral chemotherapy is given [9-11], and the incidence of TB is increasing in regions where HIV is prevalent [3]. As described in the Global Plan to Stop TB 2006-2015, management of LTBI in high HIV prevalence settings will be of paramount importance, together with DOTS expansion and provision of a tuberculosis/HIV package of prevention and care, to control and eventually to eliminate tuberculosis [12].

For several decades, identification of individuals with LTBI has been conventionally performed using the Tuberculin Skin Test (TST) [13]. Several studies have demonstrated its prognostic value with correct correlation between a positive test and potential occurrence of active TB[14-16]. Several prospective studies using TST have established the benefit of prophylactic therapy in individuals with LTBI, either in a setting of HIV co-infection or not [17-19]. However, accumulated experience has led to a full understanding of the limitations and drawbacks of TST. The limitations of TST in this setting is the need for two visits [20], the subjective nature of the test placement and its interpretation, and false-positive reactions due to Bacille Calmette-Guérin (BCG) vaccination and exposure to non tuberculous mycobacteria (NTM) [13]. In addition, HIV-infected patients have a higher rate of specific and non-specific unresponsiveness (anergy), particularly with advanced immunosuppression [21-24]. Thus, new tests for diagnosing LTBI are warranted, and ideally such tests should have several characteristics: a high sensitivity in all populations at risk, and a high specificity, regardless of BCG vaccination or infection with environmental NTM. It should also be reliable, stable over time, and with objective criteria for a positive result. Because, most of the LTBI occurred in high incidence, resource-limited countries; this test should also be inexpensive and easy to perform.

Presently, two *in vitro* assays called IFNγ-released assays [IGRAs; QuantiFERON-TB Gold^®^ *in Tube* (QF-TB-IT, Cellestis, Australia) and T-SPOT-TB^®^ (Oxford Immunotec, UK)] have been developed and are commercially available. They measure the T-cell IFNγ production after 16 to 24h of contact with *M. tuberculosis* specific antigens coded by the region of deletion 1 or RD1 (ESAT-6 + CFP-10 for T-SPOT-TB, and ESAT-6 + CFP-10 + Tb7.7 for QF-TB-IT). Both assays have a negative and a positive control, the latter being represented by the T-cell response towards the phytohemaglutinin PHA. Both assays, in addition to TST, are the only available assays for the diagnosis of *M. tuberculosis* infected individuals.

Comparison between IGRAs and TST has been performed, either in well defined active TB patients to establish their respective sensitivity or in well defined controls without any contact risk with a TB index case to establish their respective specificity. As is clearly evident, we do not benefit from a gold standard to define such a control population to compare with active TB patients where a positive culture represents the gold standard.

To deal with LTBI diagnosis, investigators have used two approaches. In the first one, investigators compared directly the results of the TST with those of the IGRAs and calculated a degree of agreement (using the Cohen’s kappa coefficient). With the second approach, investigators designed studies to establish the extent of which the test performance fits with a defined attribute (e.g., the likelihood of infection based on clinical or epidemiological characteristics). This second approach is consistent with a causal relationship and thereby strengthens the assessment of the validity (e.g., the gradient of exposure as an indicator of the likelihood of recent LTBI).

Several recent reviews have shown considerable promise for IGRA as a new diagnostic tool for LTBI in immunocompetent TB infected individuals [25-30]. IGRAs when compared to TST do not cross-react with immune responses elicited by BCG vaccination and NTM exposure. In addition, IGRA results have shown a higher sensitivity than TST in low endemic TB countries. Recent meta-analysis has further emphasized these results with suboptimal sensitivities (TST: 71%; QF-TB: 76%; ELISPOT: 88%) and high pooled specificities (TST: 66%; QF-TB: 97%, ELISPOT: 92%) [31]. Both assays were more specific than TST in samples from individuals vaccinated with BCG. Discordant TST and IGRAs reactions were frequent and largely unexplained (definitions used for the positive test results, the study population, the sample sizes, and the study design) [32-34].

Compared to the extensive numbers of studies performed in immunocompetent individuals infected with *M. tuberculosis*, there are only limited data describing IGRA performance in HIV-infected individuals, who’s immunological impairment may affect the performance of these lymphocyte-based assays. It is the purpose of this review to analyze the published studies relative to the usefulness and limitations of the IGRA in HIV infected individuals in comparison with the TST.

## REVIEW

### 1. New T-Lymphocyte-Based Tests for LTBI

#### 1.1. The IGRA Performances in HIV Infected Individuals

The first study was done in Zambian HIV-positive [35]. An “in-house” PPD and RD1 antigens ELISPOT was compared to TST in 39 HIV-TB coinfected patients (with advanced immunosuppression), 11 HIV-negative patients with active TB disease and in the 54 HIV-negative asymptomatic Zambian healthy adults. PPD and RD1 ELISPOT gave identical results in HIV uninfected patients (100%, Table **1**); but gave fewer positive responses in HIV- positive co-infected TB patients. RD1 ELISPOT positive responses were, however, more frequent than PPD ELISPOT positive responses in the same HIV-positive co-infected TB patients (Table **1**). Finally, TST and PPD ELISPOT were more often positive than RD1 ELISPOT in healthy control individuals (Table **1**) [35]. Of note, three RD1 ELISPOT positive, HIV-positive but TB negative were PPD ELISPOT and TST negative [35]. IGRAs might perform well in HIV-infected patients even better as compared to TST. However, this “in-house” assay was performed in a relatively low number of patients and the CD4+ cell counts per patient were not reported.

Following this study, several recent reports evaluated IGRAs performance in HIV- infected patients with active TB (Table **2**) [36-38]. The prevalence of the TST positive responses was always lower than the proportion of IGRA positive responses in HIV-infected patients with active TB. In this group of patients, the performances of the T-SPOT and QF-TB assays were identical showing 85 to 90 % of positive responses.

In HIV-infected patients without any TB disease, IGRAs results were more variable and might reflect the different TB epidemiological situations in the countries where the studies were performed. Higher positive responses were observed in patients from South Africa compared to European patients.

Twice the number of published studies described specific results that were obtained in individuals with LTBI comparing the respective prevalence of positive results in TST with those with IGRAs, half being evaluated in low endemic regions, the other half in high endemic regions (Table **3**) [39-44].

In three large cohorts performed in low endemic countries, data showed that only a limited number of HIV-infected individuals had positive IGRA (4.1 to 8.5%), and almost 80% of these individuals with IGRA positive results had risk factors for LTBI (history of exposure, long term residency in a high endemic country) [39-41]. When tested simultaneously, TST and QF-TB-*IT* gave comparable positive results.

In the three studies performed in high endemic countries, a higher frequency of IGRA and TST positive results was observed related to higher transmission rate of *M. tuberculosis* in this population [42-44]. TST positive results ranged from 83% (in HIV-negative LTBI individuals) to 21.4% (HIV-positive LTBI individuals). In the study performed by Rangaka and coworkers, significantly fewer (p<0.01) TST positive results were obtained in the HIV-infected group as compared to the HIV-uninfected group regardless of TST cutoff [42]. A similar decrease in positive TST results in HIV-infected individuals with LTBI was shown recently in India [45]. In comparison, IGRA results gave more homogeneous results. When the 2 IGRAs were tested simultaneously, higher numbers of individuals scored with a T-SPOT positive response as compared to QF-TB-G, in HIV-infected (52% *vs* 43%) and HIV-non infected individuals (59% *vs* 46%, respectively). However, such differences were not statistically significant (p=0.07) [42].

#### 1.2. Stratification of Results in HIV-Infected Individuals by CD4+ Cell Count

Both IGRAs depend predominantly on the CD4+-T-cells recognition of *M. tuberculosis* antigens. Test performance in the HIV-infected group will be affected by the absolute CD4+-T-cells count. Similarly, the positive control (using non-specific stimulation with PHA) depends on absolute CD4+ cell count. The PHA control is indicative of the level of immunosuppression observed in the tested individual, and interpretation of IGRAs depends on its positive result. A negative PHA result will confer an indeterminate response of the IGRAs. These two aspects have been explored by stratifying the CD4+ T-cells count with IGRAs results [39-44].

**Fig. (1).**
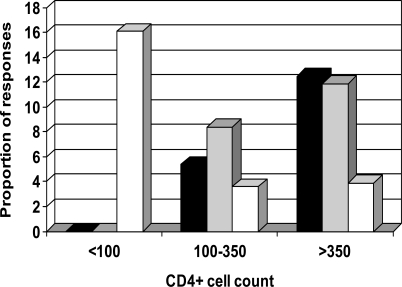
Percentage of indeterminate responses (white column) positive responders to QuantiFERON-TB-IT (black column) and to TST (grey column) stratified by CD4+ cell count. Data from Jones *et al*. [41].

**Fig. (2).**
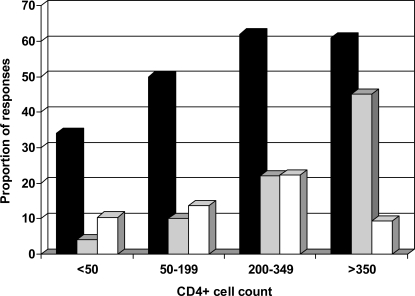
Percentage of indeterminate responses (white column) positive responders to ESAT-6/CFP10 ELISPOT (black column) and to TST (grey column) stratified by CD4+ cell count. Data from Karam *et al*. [43].

Variable percentages of indeterminate results were noticed according to the CD4+ cell count using QF-TB-*IT* [39-41]; with a higher proportion of indeterminate results in patients with low CD4+ cell counts. In individuals with CD4+ cell count lower than 100 cells/µL proportion of indeterminate results were 24%, 16%, and 37% in these three studies, respectively (Fig. **1**) [39-41]. In studies using the T-SPOT-TB, fewer indeterminate results were observed. Numbers of interpretable T-SPOT-TB assays seemed less influenced by the CD4+ cell counts (Fig. **2**), as already described by Dedha *et al. * [46].

For this reason, enhanced performances in a higher proportion of tuberculosis identified infected subjects in the lower CD4+ cell count group was observed for T-SPOT-TB as compared to QF-TB-G or QF-TB-*IT * (Figs. **1**,**2**).

In conclusion, commercially available IGRAs have evolved rapidly over the past decade. The latest generation of QF-TB-IT uses more specific antigens and is simpler to perform. In our review, the different studies performed in HIV-positive patients showed that, in contrast to the TST, HIV infection does not appear to substantially undermine the IGRA responses in patients with active TB [36-38] or LTBI [39-44]. However, the proportion of both QF-TB and T-SPOT-TB positive results decreases with advanced immune suppression; such a decline seems to be more important for QF-TB than for the ELISPOT assay. Moreover, the proportion of indeterminate results increases with advanced immune suppression and again is more likely with QF-TB-IT than with T-SPOT-TB. Because lymphopenia is often found in advanced HIV-associated immunodeficiency [47], a normalized input of PBMC used in ELISPOT assays may partly explain why this assay appears to retain its efficiency among patients with low CD4+ cell counts. The other commercially available IGRAs (QF-TB-G, or QF-TB-IT), although producing easier logistic calculation in which the cell input is not normalized, could suffer from a high rate of indeterminate results among those HIV-infected patients with advanced immunodeficiency [39-41].

#### 1.3. Need for Further Studies

The two major limitations of these new *ex vivo* IGRAs for diagnosing LTBI are firstly, that they are not able to differentiate LTBI from active TB and secondly, that they are not predictive of the development of active TB.

Recently Rangaka and coworkers [37, 42] reported an interesting approach to better identify patients with active TB among HIV-infected patients. This method directly correlates the ELISPOT results of RD1 proteins stimulation with the CD4+ T-cell count of each single patient. Using the ratio of combined T-SPOT-TB results to CD4+ cell count the authors showed that a ratio >1 was significantly more frequently observed in patients with active TB compared to latent TB. However, recent published data seemed to contradict such a prediction showing that the indicated ratio was not able to distinguish LTBI from active TB [48].

To be able to identify those individuals who do not progress to active disease, large scale-scale cohort studies with long-term follow-up of untreated populations exhibiting positive results at baseline are required. Ongoing studies are in progress in endemic area trying to evaluate the risk for active disease associated with a particular test measure (quantitative versus dichtotomic results), as shown in uninfected HIV individuals [49,50].

#### 1.4. New Directions for New Tests

Several research groups have evaluated other RD1 or RD coded protein or peptides than those developed by the commercial companies. Different selected RD1 peptides have been developed and tested in Italy by Goletti and coworkers. The authors showed that an assay based on RD1 selected peptides has a higher diagnostic accuracy for active TB in a clinical setting compared with commercially available assays based on RD1 overlapping peptides in HIV-infected or uninfected patients [36, 51, 52].

Other developments concern antigens present at the surface of *M. tuberculosis*. One group has assessed the diagnostic potential of a novel 28-kD mycobacterial protein, the heparin-binding hemaglutinin (HBHA), previously shown to stimulate high levels of IFN-γ secretion by the peripheral blood lymphocytes of LTBI subjects in comparison with QF-TB-IT [53]. The conclusion of the authors was that the commercially available IGRA may underestimate the incidence of LTBI, whereas the use of HBHA may combine the operational advantages of IGRAs with higher sensitivity and specificity for latent TB infection [53].

Other groups have tried to measure the different cytokines (IFN-γ, IL-2, IL-10) released after stimulation with selected antigens to differentiate a population at high risk of active TB, showing a higher ratio of IFN-γ/IL-10 in protected individuals [54].

### 2. New *Ex Vivo* Cell-Mediated Killing Assay

Other researchers are looking for biomarkers for innate immunity or acquired immune protection. It has been reported that *in vitro* cell-mediated killing assays may provide a promising measure of the functional capacity of cells to kill mycobacteria [55].

Whole blood killing assays (WBKA) have the advantage of including all cells with potential anti-mycobacterial activity compared to infection of isolated differentiated monocytes and are more easily adapted for use in large scale studies. Inhibition of *in vitro* growth of the bacilli has been measured using different techniques: conventional liquid medium culture (BACTEC system, BD), or luminescent readout of reporter-gene-tagged BCG (BCG *lux*) expressing a recombinant luciferase enzyme.

Using the second methodology, a study reported an association between low CD4+ cell counts, low IFN-γ production and impaired ability to regulate growth of *M. bovis* BCG in blood from HIV-infected children in South Africa [56]. A more recent study from the same authors reported that this WBKA was able to monitor *in vitro* the anti-mycobacterial immune response of HIV-infected children during antiretroviral therapy [57]. The authors confirmed their earlier results showing that before HAART the blood from HIV-infected children demonstrated a low ability to restrict the growth of BCG in the functional WBKA; in contrast the introduction of HAART was followed by rapid and sustained reconstitution of specific antimycobacterial immune response, measured as a decrease in growth of the bacilli. This *in vitro* model mirrors the *in vivo* observation of decrease susceptibility to TB in HIV-infected adults receiving HAART [58].

### 3. Preclinical Detection of Antibody

Tests for serodiagnosis of TB, either based on ELISA or immunochromatographic strip technology, have been developed and have been evaluated in immunocompetent patients with active TB. The tests, with the best performance characteristics, have an observed sensitivity and specificity around 60% and 90%, respectively [59], demonstrating that the existing serological tests cannot be used as stand-alone diagnostic test for TB. Moreover, it has been generally observed that HIV-infected individuals with TB have suppressed antibody levels [60]. In this last study, the authors showed that while all HIV-negative and PPD-positive patients had IgG antibodies recognizing the 38-, 28-, and 19-kDa *M. tuberculosis* antigens, only 26% of those HIV-positive and PPD-positive (all with < 400 CD4+ cells/mm3) and none of the HIV-positive tuberculosis patients recognized these antigens. In an effort to increase the performance of potential serological tests, it was relevant to explore whether novel proteins or non-protein antigens specific for *M. tuberculosis* were able to significantly increase sensitivity and specificity. Several proteins, TB16.3, TB9.7, U1, and Mtb81, have been described as serodiagnostic antigens that perform as well as or even better in the HIV-infected group, as recently reviewed [61, 62].

Different authors have also shown that antibodies detecting glycolipid antigens (PGL-TB1, DAT), specific for *M. tuberculosis*, were present in HIV-TB co-infected patients with levels higher than those in HIV uninfected TB patients [63, 64]. Moreover, antibodies to two recombinant proteins (MPT51 and Mtb81), and PGLTb1 or DAT antigens have been shown to be present in serum samples obtained during sub-clinical TB in a high proportion of HIV-infected patients tested [63-65].

Although, *ex vivo* tests based on cellular immune response (IGRAs) performed in HIV-infected individuals with advanced immunodeficiency (with CD4+ lower than 100 cells / μL) have been shown to be associated with a high proportion of indeterminate results, the anti-PGL-Tb1 antibodies levels have been demonstrated to be independent of the CD4+ cell counts [63]. Thus, insight into the serological responses based on anti-glycolipid antigens elicited during sub-clinical TB could provide additional markers that identify incipient infection with *M. tuberculosis* before progression to clinical disease occurs [63, 65].

### 4. Potential New Algorithm for Detecting and Treating HIV-Infected Persons at High Risk for Progression to Active TB

**Fig. (3).**
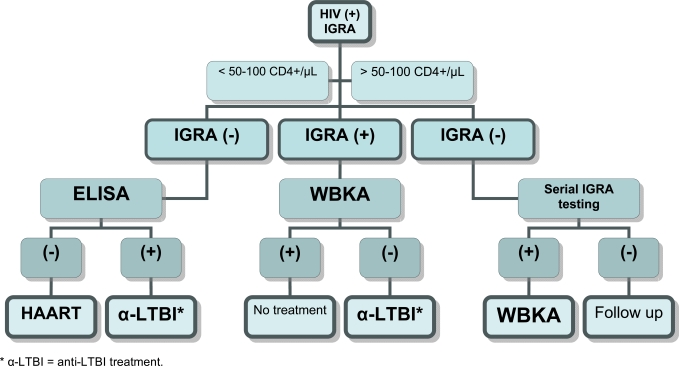
New possible algorithm for targeting HIV-infected individual establishing the highest risk group and the specific treatment chemoprophylaxis. IGRA (Interferon-gamma release assay), WBKA (Whole Blood Killing Assay), ELISA (antibody assay with PGL-Tb1).

Using the various blood assays described above, and depending on their validation in large scale longitudinal studies, a new algorithm might be considered (Fig. **3**).

In low or high endemic TB areas, all HIV-infected individuals should be first tested with IGRAs (QF-TB-IT or T-SPOT-TB) and then stratified according to their level of immuno- suppression. Those individuals with a CD4+ cell count higher than 50-100 cells/µL will potentially have either a positive or a negative IGRA response. Individuals with a negative response (meaning absence of recent TB transmission), should have serial IGRA testing at regular interval (every 6 months). Individuals with a positive response (meaning the presence of recent TB transmission) should be tested with the WBKA to evaluate their potential antimycobacterial immune protection. If a positive response (surrogate maker showing the capacity to restrict in vitro growth of M. tuberculosis or BCG) is detected, these individuals could be considered as immune and should not receive any anti tuberculous prophylaxis. If a negative WBKA response is detected, such recently infected individuals without any specific immunity should be considered for anti TB chemoprophylaxis. A certain proportion of Individuals, with < 50-100 CD4+ cell count /µL may produce an IGRA positive response and the same protocol as above should be followed for IGRA positive response. For those individuals with indeterminate or negative results with IGRA, the only suitable test that might demonstrate infection with TB is the measurement of circulating IgG anti-PGL-Tb1 antibody. Individuals with ELISA positive results should be considered at high risk and should received anti-TB chemoprophylaxis after eliminating active TB disease. A systematic approach for blood culture testing to detect circulating mycobacteria should also be considered. For those without circulating anti- PGL-Tb1 antibody should be considered for empirical HAART [66].

## CONCLUSIONS

There is a definite need to diagnose and treat LTBI in all HIV-infected individuals at high risk of progression to active TB disease. These individuals should receive anti-TB chemoprophylaxis after thorough testing to eliminate active TB disease. IGRAs based on specific RD1 peptides have shown promising results for diagnosing LTBI not only in immunocompetent individuals, but also in HIV-infected persons at higher risk for development of active disease. Although, these dichotomous (positive/negative) measures cannot distinguish between LTBI and active disease, operational research clinical studies are ongoing to demonstrate their potential role in predicting the risk of progression. Evidence coming from studies showing a high ratio of combined quantitative T-SPOT-TB results to CD4+ cell count in patients with active TB should be confirmed in different settings in order to classify patients with active TB from those with LTBI. It is thus likely that tuberculin skin testing being largely inadequate in these immunocompromised patients will be in the next future substituted for the IGRAs.

Other issues need to be resolved in relation to the identification of correlates of immune protection or “functional biomarkers” that will facilitate the rational design for drugs for the clearance of TB infection. After validation, the WBKA will find its right place among a new algorithm that might facilitate the management of HIV-infected individuals at high risk of progression. Similarly, antibody markers might predict ongoing progression from latent to active TB in HIV-positive subjects, including those with negative PPD skin test or with indeterminate IGRA results.

## Figures and Tables

**Table 1:** Frequency of Positive Results in Zambian TB Patients and Healthy Individuals According to their HIV Status Tested with TST or with ELISPOT. Data from Chapman *et al*. [35]

Patients Tested	HIV Status	TST (5 TU)	ELISPOT	
			PPD	ESAT6/CFP10
TB disease	negative positive	NT* NT*	11/11 (100%) 28/39 (72%)	11/11 (100%) 35/39 (90%)
Healthy Zambian	negative positive	28/35 (80%) 5/14 (36%)	45/54 (83%) 6/21 (29%)	37/54 (69%) 9/21 (43%)
Healthy UK**	Negative	NT*	33/40 (83%)	0/40 (0%)

* NT: non tested

** 30/48 (80%) have been BCG vaccinated.

**Table 2:** Proportion (in Percentage) of IGRA Indeterminate and Positive Results in Groups of HIV-Infected Patients with Active Tuberculosis and in HIV-Positive Patients without Tuberculosis Tested with TST, T-SPOT-TB and QuantiFERON –TB

Reference	Diagnosis	Number Subjects	Mean CD4+/µL	Tests			
				IGRA			
				TST	SPOT	QF-TB	Indeterminate
Vincenti (Italy) [36]	Active TB	45	152	47.0	84.6	84.6	20.0
	Non TB	66	236	39.9	35.7	25.0	18.2
Rangaka (South Africa) [37]	Active TB	41	167	67.0	90.0	90.0	12.2
	Non TB	41	464	51.0	75.0	79.0	7;3
	HIV (-) controls	41	NA*	69.0	83.0	83.0	0.0
Clark (UK) [38]	Active TB	154	209	NT**	90.3	NT**	4.5
	LTBI	47	294	NT**	20.0	NT**	4.5

* NA: non applicable.

** NT: non tested.

**Table 3:** Proportion (in Percentage) of IGRA Indeterminate and Positive Results in Cohorts of HIV-Infected Patients with Latent Tuberculosis Infection and in HIV-Negative Control Patients Tested with TST, T-SPOT-TB and/or QuantiFERON –TB in Low or High Endemic Tuberculosis Areas

Reference	Subjects	Number	Mean CD4+/µL	TST (%)	IGRA Results		
					T-SPOT	QF-TB	Indeterminate
Brock (Denmark) [39]	cohort	590	523	NT	NT(a)	4.1	3.4*
Luetkemeyer (USA) [40]	cohort	294	132	9.3	NT(a)	8.5*	5.1*
Jones (USA) [41]	cohort	201	453	6.8	NT	5.8	4.9*
Rangaka (South Africa) [42]	HIV(+)	74	392	52*	52.0	43.0	7.0*
	HIV(-)	86	NA(b)	83	59.0	46.0	2.0
Karam (Senegal) [43]	cohort	285	180	21.4*	50.6*	NT	13.3*
Lawn (South Africa) [44]	cohort	40	114	43	62.0	NT	10.0

(a); NT: non tested; (b); NA: non applicable.

* CD4+ cell counts dependent frequency.
